# Manejo desde atención primaria de la infección por la viruela del mono (MPOX) en humanos

**DOI:** 10.1016/j.aprim.2023.102680

**Published:** 2023-06-19

**Authors:** Javier Arranz Izquierdo, José María Molero García, María Isabel Gutiérrez Pérez

**Affiliations:** a Instituto de Investigación Sanitaria de Illes Balears (Idisba), Illes Balears, España; b Centro de Salud Escola Graduada, Ibsalut, Palma de Mallorca, Illes Balears, España; c Grupo de trabajo de enfermedades infecciosas de SemFYC, Madrid, España; d Centro de Salud San Andrés, DA Centro (SERMAS), Madrid, España; e Centro de Salud Delicias I (GAP Oeste), Valladolid, España

**Keywords:** Viruela del simio, Medicina de familia, Tratamiento, Vacuna de la viruela, Simian smallpox, Family medicine, Treatment, Smallpox vaccine

## Abstract

La viruela del mono (MPOX) es una zoonosis vírica endémica en países de África occidental o central que esporádicamente se exporta a otras regiones. En mayo del 2022, comenzó a ocurrir un brote mundial de viruela MPOX en varias naciones de Europa y Norteamérica. La mayoría de los casos notificados se identificaron a nivel ambulatorio y afectaron principalmente a hombres que tienen sexo con hombres (HSH). El contagio es por contacto estrecho con lesiones, líquidos corporales, secreciones respiratorias o con material contaminado, de persona o animal infectado. El cuadro clínico es similar a la viruela humana, con menor gravedad. Predomina la afectación cutánea leve y autolimitada tras dos a cuatro semanas. En HSH aparecen lesiones cutáneas atípicas debido a la manera de contagio. En ciertos grupos de riesgo pueden presentarse formas graves o complicaciones. La tasa de letalidad es de 3 a 6% según el clado responsable. El diagnóstico de sospecha se confirma con la detección del virus, a partir de exudados de las lesiones o costras, con técnicas de amplificación de ácidos nucleicos mediante reacción en cadena de la polimerasa (PCR) convencional o en tiempo real. El manejo clínico en la mayoría de los casos se realiza desde atención primaria (AP), mediante el control de los principales síntomas. Entre 5 a 10% requieren un manejo hospitalario y existen algunas opciones de tratamiento antiviral específico. Las vacunas frente a la viruela humana protegen contra la MPOX y se utilizan como profilaxis pre y posexposición a personas de riesgo. Las medidas para reducir la exposición al virus, es la principal estrategia de prevención de la MPOX. Además, el papel del médico de familia es clave para controlar la propagación del virus de la MPOX mediante la vigilancia activa y el diagnóstico temprano de la enfermedad.

## Abstract (en)

Monkeypox (MPOX) is a viral zoonosis endemic in West or Central African countries that is sporadically exported to another area. In May 2022, a global outbreak of MPOX smallpox began to occur in several countries in Europe and North America. Most of the reported cases are identified at the outpatient level and mainly affect men who have sex with men (MSM). Transmission is by close contact with lesions, body fluids, respiratory secretions or contaminated material from an infected person or animal. The clinical picture is similar to human smallpox, with less severity. Mild, self-limiting skin involvement predominates after 2-4 weeks. In MSM, atypical skin lesions appear due to the mode of infection. Severe forms or complications may appear in certain risk groups. The case fatality rate is 3%-6% depending on the clade responsible. The diagnosis of suspicion is confirmed by detection of the virus from exudates of lesions or scabs, with nucleic acid amplification techniques by conventional or real-time PCR. Clinical management in most cases is performed in primary care (PC), by monitoring the main symptoms. Between 5-10% require hospital management and there are some specific antiviral treatment options. Human smallpox vaccines protect against MPOX and are used as pre- and post-exposure prophylaxis for persons at risk. Measures to reduce exposure to the virus are the main MPOX prevention strategy. In addition, the role of the family physician is key to controlling the spread of MPOX through active surveillance and early diagnosis of the disease.

## Introducción

La viruela del mono o de los simios (MPOX) se detectó por primera vez en humanos en 1970 y desde entonces ha tenido una evolución endémica hasta en 11 países de África occidental y central. Esporádicamente, se habían detectado casos y brotes fuera de estas zonas endémicas^1^. Desde mayo del 2022 se ha notificado un número significativo de casos en varios países donde la enfermedad no es endémica, principalmente Europa y Norteamérica y, además, continúan presentándose en las zonas endémicas^1^. Por ese motivo se convirtió en la séptima Declaración de Emergencia de Salud Pública de Preocupación Internacional (PHEIC) con más de 80.000 casos y 141 fallecimientos, registrados en 111 países^1^, ^2^, finalizando como tal PHEIC el 11 de mayo de 2023.

La MPOX es una zoonosis causada por un *Orthopoxvirus* del mismo género que la *Variola* y los virus *Vaccinia*. Fue descubierta en 1958. Existen dos cepas o clados, una responsable de la enfermedad en el África central y otra, de menor severidad, detectada en África occidental. La cepa del brote actual estaría relacionada con esta última, compartiendo características con el virus causante del brote de 2018-19 en Nigeria. Los datos de la secuenciación inicial indican que hay más mutaciones de las esperadas en su ADN, lo cual plantea la posibilidad de que el virus circulante está experimentando una adaptación humana acelerada^3^,

El periodo de incubación de la MPOX abarca entre seis y 16 días. En el brote actual podría situarse en los 8-9 días^4^, ^5^. El intervalo de serie (tiempo entre el inicio de síntomas en el caso primario y en el secundario) es algo mayor que el periodo de incubación, ocho a nueve días, si bien algunos autores^5^ apuntan a que en un porcentaje elevado de casos el intervalo de serie podría ser inferior, lo que indicaría la posibilidad de transmisión en el periodo presintomático. En este mismo sentido, un reciente estudio prospectivo, informa de la detección de ADN viral cuatro días antes de presentar síntomas en cinco de seis (83,3%) pacientes, confirmando asimismo la viabilidad del virus^6^.

La transmisión de la MPOX requiere de un contacto cercano con la fuente, pudiendo ser por medio de fluidos o superficies contaminadas, persistencia de más de 15 días tras un caso índice^7^, por contacto directo con lesiones de MPOX o por contacto estrecho persona-persona.

Hasta la fecha no hay datos para pensar que la transmisión única persona-persona pueda sostener la enfermedad en el tiempo.

**Figura 1:**
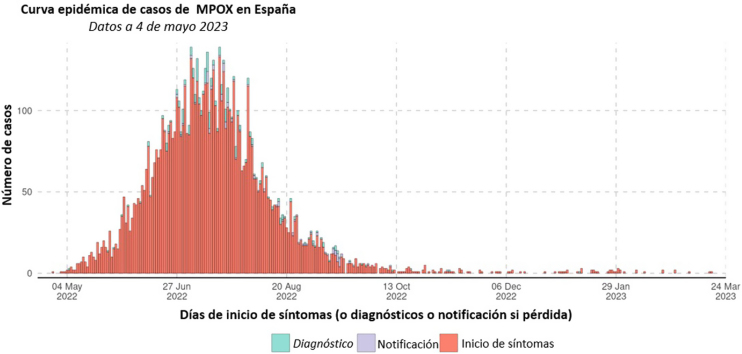
Curva epidémica, nuevos casos de viruela del mono en España. Elaborado a partir de los datos obtenidos de la situación epidemiológica de la MPOX notificada a la OMS^1^.

España ha sido uno de los países con mayor número de casos notificados en el mundo, sobre todo al inicio del actual brote, con una cifra de 7.555 al 23 de mayo de 2023^8^, siendo la tendencia actual claramente descendente, si bien existe un retardo entre el inicio de los síntomas y la notificación de seis días y de siete para el diagnóstico de confirmación (fig. 1). Las comunidades autónomas más afectadas son Madrid con 2.552 casos; Cataluña con 2.327; Andalucíacon 884 y Comunidad Valencianacon 557 casos^8^. En el brote actual de nuestro país, 97,8% de los casos detectados son hombres, la mediana de edad está en los 37 años (el 66,8% entre 30 y 49). De los casos diagnosticados, 21,1% informaron haber tenido contacto con un caso probable o confirmado. De forma similar al resto de los países afectados, 76,9% eran hombres que habían tenido relaciones sexuales con otros hombres (HSH). La comorbilidad más frecuentemente reportada es la infección por virus de la inmunodeficiencia humana (VIH) en 2.833 de 6.988 (40,5%) de casos con información al respecto^8^.

Aunque se trata de un número muy limitado de casos, algunos autores han planteado la posibilidad de reinfecciones^9^.

## Diagnóstico clínico y microbiológico

### Diagnóstico clínico

El conocimiento sobre las manifestaciones clínicas es limitado. La MPOX puede presentarse con malestar general (fiebre, escalofríos, linfadenopatías, mialgias, fatiga, dolor de cabeza y espalda), seguido de un exantema característico^4^, ^10^, ^11^, ^12^ (tabla 1). Esta erupción puede aparecer desde uno a dos días antes hasta tres a cuatro días después de la clínica sistémica, comienzan en la zona de la cabeza, extendiéndose hacia el tronco y las extremidades. Las lesiones iniciales son máculas (2 a 5 mm) que evolucionan a pápulas, vesículas y pseudopústulas (vesículas con restos celulares, sin pus). Aproximadamente una semana después de su aparición, las pústulas pasan a una fase de costra, se secan y caen (entre siete a 14 días después del inicio del exantema)^10^. En ocasiones pueden persistir cicatrices^10^.

**Tabla 1:** Criterios para la clasificación de los casos de viruela del mono (Monkeypox)

Criterio clínico	Persona con un cuadro clínico altamente sugestivo de infección por MPOX en la que se han descartado o el diagnóstico diferencial indica que hay muy baja sospecha de otras patologías. Exantema vesicular o pustular (especialmente si es umbilicado) en cualquier parte del cuerpo con uno más de los siguientes: fiebre (> 38,5 °C), dolor de cabeza intenso, mialgia, artralgia, dolor de espalda, linfadenopatía.
Criterio epidemiológico	Si en los 21 días antes del inicio de síntomas cumple uno de los siguientes: • Ha tenido un contacto estrecho con un caso confirmado o probable de MPOX • Ha mantenido relaciones en contextos sexuales de riesgo • Tiene historia de viaje a zonas endémicas de África occidental o central en los que se ha identificado circulación del virus
Criterio de laboratorio	Detección de genoma de virus MPOX mediante PCR específica o genérica para *Orthopoxvirus* en muestra clínica

Adaptada de Vallejo et al.^12^

MPOX: viruela del mono (monkeypox); PCR: reacción en cadena de la polimerasa.

Caso sospechoso*: cumple el criterio clínico.

Caso probable*: cumple criterio clínico y criterio epidemiológico.

Caso confirmado: cumple criterio de laboratorio.

Caso descartado: casos sospechosos o probables en los que el resultado de laboratorio. en muestras de alta calidad ha sido negativo.

*Se considerarán casos en investigación.

La presentación clínica en el brote actual difiere de los casos endémicos. En España, donde la mayoría de los contagios se han producido entre HSH, los infectados han presentado lesiones cutáneas-mucosas y complicaciones relacionadas con el área de inoculación, como genitales, perianales y orales, incluidas proctitis, amigdalitis y faringitis ulcerosa, generalmente sin afectación sistémica^4^, ^11^. Aunque las linfadenopatías locales estaban presentes, no se observó inflamación generalizada^4^. Los signos y síntomas más frecuentes reportados en mujeres fueron síntomas generales, exantema en diferentes localizaciones y adenopatías locales. El exantema es más recurrente en mujeres, aunque la localización anogenital es significativamente menos común que en el hombre^12^. No se encontraron diferencias con el hombre en el número de casos con adenopatías^12^.

Otras manifestaciones menos frecuentes consisten en infecciones oculares (conjuntivitis, blefaritis, queratitis o pérdida de visión) y otras lesiones genitales, perianales o faciales con evolución a gran placa coalescente, ulceración o costra y celulitis superpuesta^4^, ^11^, ^12^. También puede cursar con lesiones intestinales exudativas o con edema que producen obstrucción, edema prepucial que resulta en parafimosis o cuadros de encefalomielitis^4^, ^11^, ^12^.

Debido a manifestaciones cutáneo-mucosas menos típicas, relacionadas con la vía de transmisión sexual, el diagnóstico de sospecha es más difícil y se debería tener un alto índice de sospecha de la enfermedad, particularmente en personas con exposición potencial.

### Diagnóstico de laboratorio

#### Pruebas de análisis clínicos

Determinados hallazgos pueden ser signos de una enfermedad grave o complicada como leucocitosis y/o trombocitopenia (más de un tercio de infectados), aumento de transaminasas, hipoalbuminemia y la elevación de urea^10^.

#### Pruebas microbiológicas

La confirmación microbiológica en los casos con sospecha de infección debe realizarse lo antes posible. En la actualidad, las pruebas disponibles para el diagnóstico son:

a) Pruebas de detección viral. La más utilizada es la amplificación de ácidos nucleicos, que emplea la prueba de la reacción en cadena de la polimerasa (PCR) convencional o en tiempo real específica para el virus de la MPOX a partir de exudados de las lesiones o de las costras, según la fase de la erupción^10^, ^13^, ^14^, ^15^. Las mejores muestras son las lesiones vesiculares. Las muestras sanguíneas son poco rentables por la menor probabilidad de viremia tras la aparición de los síntomas^14^. La muestra de frotis nasofaríngeos son poco útiles para el diagnóstico primario^14^. La detección positiva mediante una PCR para el virus de la MPOX en casos sospechosos, indican la confirmación de la infección por este virus^14^. Ante resultados positivos, con baja carga viral, se recomienda repetir la prueba si la clínica es atípica o el grado de exposición es bajo. La determinación se sugiere para los contactos de alto riesgo de un caso confirmado o altamente probable que hayan desarrollado síntomas sistémicos sin afectación cutánea^14^, ^15^.
b) Las pruebas serológicas no permiten el diagnóstico rápido de la infección aguda. Pueden tener utilidad con fines epidemiológicos para respaldar el diagnóstico cuando no se pueden realizar pruebas virales o si la PCR no ofrece resultados concluyentes^10^, ^14^, ^15^. La vacuna puede interferir con las pruebas serológicas^14^.
c) En el brote actual en España, donde la mayoría de los casos eran HSH con comportamiento sexual de alto riesgo, la coinfección con otras infecciones de transmisión sexual (ITS) fue frecuente^4^, ^11^. En estos pacientes, podría estar justificado el despistaje de ITS en función del riesgo (tabla 2).

**Tabla 2:** Factores de riesgo epidemiológicos de los casos de viruela del mono (Monkeypox)

Edad	La mediana de edad de los pacientes en todo el mundo es de 35 años; los pacientes varones de 18 a 44 años representan 79,5% de los casos
Etnia/raza	• Los datos que existen son de EE. UU. y faltan en muchos casos (34,7; 29,8% blancos; 29,7% hispanos; 3,3% asiático-americanos y menos de 3% de otra etnia /raza)
Actividad sexual	• La mayoría de los casos en el brote actual de 2022 en todo el mundo han sido en hombres que se identifican como homosexuales o bisexuales o en otros hombres que tienen sexo con hombres (HSH) • No se considera enfermedad de transmisión sexual, pero, se puede propagar a través del contacto directo con piel durante la actividad sexual (incluidos besos, caricias, sexo oral y compartir ropa de cama y prendas de vestir) • Tener múltiples contactos sexuales o parejas aumenta el riesgo de exposición e infección
ITS	• Se han informado de infecciones transmisión sexual concomitantes o en el año anterior en los pacientes con viruela del mono.
VIH	• Entre los casos del brote de 2022 a nivel mundial, 45% han sido positivos para el VIH; las tasas han oscilado entre 28 y 51% • Se desconoce en este momento si el VIH *per se* aumenta el riesgo de infección por viruela del mono
Profesional sanitario	• Tocar la piel, las lesiones cutáneas o los fluidos corporales de un paciente • Tocar materiales contaminados (ropa de cama o vestimenta) • Permitir que su propia ropa sin protección toque la piel del paciente, lesiones en la piel, fluidos corporales o materiales contaminados • Estar dentro de la habitación de un paciente o cerca de un paciente durante procedimientos que generan aerosoles • Presencia cercana y prolongada cerca de un paciente
Público	• Los contactos personales y domésticos también corren riesgo a través del contacto directo e indirecto con lesiones, material de la lesión, secreciones respiratorias, otros fluidos corporales y superficies o materiales contaminados (p. ej., platos, utensilios, ropa y ropa de cama)

Adaptada de Instituto de salud Carlos III^13^ y CDC^15^.

ITS: infecciones de transmisión sexual; VIH: virus de la inmunodeficiencia humana.

## Valoración clínica, situaciones especiales, criterios de derivación

### Valoración inicial del paciente con sospecha de MPOX

Aunque la mayoría de las personas con MPOX presentan una enfermedad leve y se recuperan espontáneamente y de forma completa en dos a cuatro semanas (más de 90%), se han observado otros casos que cursan con complicaciones o evolucionan hacia formas graves y potencialmente mortales (tabla 3)^15^, ^16^, ^17^. Solo 245 casos han requerido ingreso hospitalario en nuestro país^7^. Las complicaciones graves pueden incluir neumonía, sepsis, encefalitis y pérdida de la visión debido a una infección y posterior cicatriz corneal^15^, ^18^. Los casos graves están relacionados con el grado de exposición al virus y la vulnerabilidad de la persona. Se ha identificado un grupo de individuos con alto riesgo de complicaciones incluidas las formas más severas de la infección (tabla 4)^15^, ^16^, ^17^.

**Tabla 3:** Complicaciones de la infección por la viruela del mono en humanos

**Locales** • Dolor asociado con las lesiones de piel y mucosas • Sobreinfección bacteriana de lesiones de la piel y mucosas^a^ (celulitis, abscesos, piomiositis o infección necrosante de tejidos blandos) • Linfadenopatía cervical^a^ que puede complicarse con absceso retrofaríngeo y causan disfagia o dificultad respiratoria • Exfoliación extensa de la piel^a^ • Lesiones oculares^b^ • Complicaciones amigdalinas^c^ (amigdalitis, celulitis periamigdalina, absceso amigdalino/periamigdalino y amigdalitis necrosante) • Complicaciones anorrectales^c^ (abscesos perianales, proctitis, perforación rectal y obstrucción) • Complicaciones genitales^c^ (obstrucción o estenosis de prepucio del pene, vulva, vagina y uretra)
**Generales:** • Deshidratación grave^d^, alteraciones electrolíticas o shock hipovolémico^c^ • Malnutrición^d^ • Bronconeumonía bacteriana • Síndrome de dificultad respiratoria aguda (SDRA) • Sepsis o shock séptico • Neurológicas (encefalitis, mielitis transversa y convulsiones) • Complicaciones obstétricas (muerte fetal o el aborto espontáneo) • Fallecimiento (0-3,6%)

Fuente: Elaboración propia a partir de CDC^15^, WHO^16^ y Heymann^17^.

a Mayor frecuencia.

b Infección corneal que puede provocar úlceras/cicatrices en la córnea y pérdida permanente de la visión.

c Probablemente cicatrices secundarias o estenosis debido a lesiones en la vía de inoculación.

d Multicausal: limitación en la ingesta/deglución por lesiones bucofaríngeas, fiebre, vómitos, diarrea, acumulación subcutánea de fluidos en la fase de costra y exfoliación extensa de la piel.

**Tabla 4:** Factores de riesgo y signos clínicos que están asociados a enfermedad grave y evolución clínica desfavorable

Grupos de pacientes con mayor riesgo de presentar un cuadro grave o complicaciones	• Niños (particularmente < 8 años) • Mujeres embarazadas o lactantes • Personas con inmunosupresión grave^a^ • Personas con enfermedades cutáneas agudas o crónicas que afectan la integridad de la piel^b^
Signos y síntomas clínicos de complicaciones (en cualquier momento de la valoración clínica)	• Infección bacteriana de los tejidos blandos (induración, calor, empeoramiento del dolor, secreción purulenta, edema, crepitación, flujo maloliente o reaparición de fiebre) • Fiebre persistente (> 5 días) o reaparición a lo largo de la evolución • Náuseas y vómitos incoercibles • Disminución de la ingesta oral, intolerancia a la ingesta oral o ingesta deficiente • Signos de deshidratación • Linfadenopatía cervical dolorosa que causa disfagia (riesgo de absceso retrofaríngeo o compromiso respiratorio) • Dolor ocular y/o alteraciones de la visión • Hepatomegalia • Dificultad respiratoria/neumonía • Signos de sepsis • Mareos o estado mental alterado (desde confusión a obnubilación) • Hipotensión mantenida o shock hipovolémico
Índice de gravedad de las lesiones cutáneas^c^	• Leve (< 25 lesiones cutáneas) • Moderada (25–99 lesiones cutáneas) • Grave (100–250 lesiones cutáneas) • Muy grave (> 250 lesiones cutáneas)
Alteraciones analíticas sugerentes de gravedad	• Hipertransaminasemia • Nitrógeno ureico en sangre (BUN) bajo • Hipoalbuminemia • Leucocitosis • Plaquetopenia
Empeoramiento clínico durante el seguimiento	• Empeoramiento intenso del estado general, debilidad o astenia • Reaparición de la fiebre • Empeoramiento de las lesiones (aumento del número, enrojecimiento rápido de la piel y signos de sobreinfección bacteriana) • Incapaz de comer y beber, pérdida de peso, signos de desnutrición (p. ej. atrofia muscular, edema nutricional, etc.) • Sospecha de neumonía grave o afectación neurológica (confusión y cefalea intensa) • Baja producción de orina

Fuente: modificada de CDC^15^, WHO^16^ y Heymann^17^.

a Incluye afecciones por infección del VIH avanzado o mal controlado, leucemia, linfoma, malignidad generalizada, trasplante de órganos sólidos, terapia con agentes alquilantes, antimetabolitos, radiación, inhibidores del factor de necrosis tumoral, corticoterapia a dosis altas, receptor de un trasplante de células madre hematopoyéticas < 24 meses después del trasplante o ≥ 24 meses pero con enfermedad de injerto contra huésped o recaída de la enfermedad, o tener una enfermedad autoinmune con inmunodeficiencia como componente clínico.

b Incluye la presencia de dermatitis atópica u otras afecciones o infecciones exfoliativas activas de la piel (psoriasis, enfermedad de Darier o queratosis folicular, eczema, impétigo, varicela primaria, zoster o herpes).

c Basado en la experiencia de la viruela.

**Figura 2:**
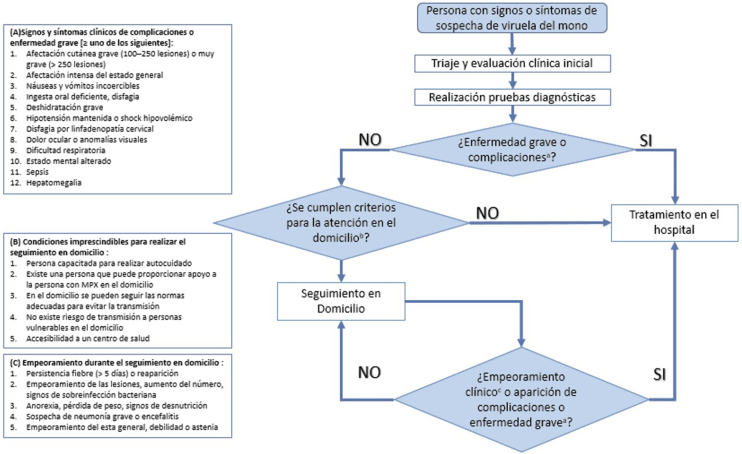
Manejo de casos de viruela del mono (Monkeypox).

En la evaluación clínica inicial de todos los pacientes con sospecha o confirmados de MPOX se deben determinar la presencia de factores de riesgo para complicaciones o enfermedad grave y la aparición de signos y síntomas de aquellos que muestren un tipo de enfermedad grave o complicaciones (tabla 4, fig. 2)^15^, ^19^, ^16^, ^17^, ^18^. La tasa de letalidad de MPOX ha oscilado entre 0 y 11% en la población general de los países africanos, y es más alta entre los niños pequeños^13^. La tasa de letalidad del clado que circula actualmente en España (África occidental) es muy baja en adultos, inferior a 1%, frente a 10% del clado de África central^13^.

Los pacientes con infección MPOX sospechosa o confirmada leve y sin complicaciones puedan aislarse en casa durante el periodo infeccioso, hasta que ya no sean contagiosos^13^, ^17^, ^18^. El seguimiento de estos casos se podrá realizar por teléfono, telemedicina o por correo electrónico. La periodicidad dependerá de los hallazgos de la evaluación inicial y de la evolución del estado de salud. En personas con factores de alto riesgo de complicaciones se realizará un seguimiento más estrecho para detectar los signos y los síntomas de complicaciones o de deterioro clínicos (tabla 4)^15^, ^16^, ^17^. El juicio clínico sigue siendo fundamental en las valoraciones durante el seguimiento.

### Situaciones especiales/consideraciones para ciertas poblaciones

Existen determinados grupos de personas donde la infección MPOX presenta elementos característicos y diferenciadores que condicionan el manejo clínico^15^, ^16^, ^17^.

a) **Niños.** La presentación clínica de niños y adolescentes es similar a la de los adultos, aunque se han informado complicaciones graves en recién nacidos, lactantes y niños menores de ocho a 10 años, en niños con eczema y otras afecciones de la piel e inmunodeprimidos.

Actualmente, no se conocen bien las posibles complicaciones, aunque la infección puede cursar con encefalitis, celulitis, neumonía, sepsis, abscesos, obstrucción de las vías respiratorias debido a linfadenopatía grave, queratitis y cicatrización de la córnea.

b) **Personas inmunocomprometidas** (incluidas aquellas con infección por VIH avanzada o no tratada), pueden presentar una erupción atípica, como una erupción diseminada o una enfermedad más grave (p. ej., sepsis, erupción cutánea diseminada, enfermedad hemorrágica, gran número de lesiones confluentes o parcialmente confluentes, lesiones necróticas, linfadenopatía grave, infecciones y otras complicaciones que requieran hospitalización).

No se ha demostrado que la infección por VIH aumente el riesgo de MPOX después de la exposición. El curso en la mayoría de los casos es relativamente leve, similar a los pacientes seronegativos para el VIH. No obstante, los casos con VIH avanzado sin control adecuado (CD4<200 células/μL), pueden tener un mayor riesgo de enfermedad grave o prolongada.

Con mayor frecuencia, la presentación clínica es atípica (ausencia de fiebre y lesiones anogenitales más notables, además es más probable la presencia de una erupción confluente o parcialmente confluente, tasas más altas de infección bacteriana secundaria y una enfermedad más prolongada (y, por lo tanto, también un periodo más largo de infecciosidad).

c) **Mujeres embarazadas y puerperio**. Los datos sobre la infección MPOX en el embarazo son limitados. Se desconoce si las mujeres embarazadas son más susceptibles a la infección o si esta es más grave durante la gestación.

La presentación clínica no se diferencia de las personas no embarazadas y la mayoría de los casos consisten en una enfermedad leve y se recuperan espontáneamente. No obstante, existe un riesgo de transmisión al feto durante la gestación o al recién nacido por contacto cercano durante y después del parto, y el riesgo de infección grave en los recién nacidos. Además, hay mayor probabilidad de aborto, partos prematuros y muerte fetal.

Por este motivo las personas embarazadas se consideran un grupo de alto riesgo de la infección por MPOX. La Organización Mundial de la Salud (OMS) solo recomienda inducir el parto o recurrir a la cesárea en los casos en que esté médicamente justificado y en función del estado de la madre y el feto.

Debe vigilarse estrechamente a los lactantes recién nacidos de madres con MPOX en busca de evidencia de posible exposición o infección congénita o perinatal. Los lactantes o niños de corta edad también pueden estar expuestos a través del contacto estrecho. De igual manera, se debe valorar la posibilidad de interrumpir la lactancia materna en el caso de una madre infectada en función del estado físico general de esta y la gravedad de la enfermedad.

d) **Poblaciones sexualmente activas**. Los pacientes deberían abstenerse de mantener relaciones sexuales hasta que finaliza el periodo de contagiosidad. La OMS recomienda usar preservativo de forma constante durante las relaciones sexuales (penetración incentiva y receptiva oral/anal/vaginal), durante 12 semanas tras la recuperación para evitar la transmisión potencial de la MPOX^6^.

### Criterios de derivación a atención hospitalaria

La mayoría de las personas con MPOX tienen un curso autolimitado y se recuperan, manteniéndose en el ámbito comunitario. No obstante, entre 5-10% requieren un manejo hospitalario^13^, ^15^, ^17^. Los pacientes con enfermedad grave o en presencia de algunas complicaciones (Tabla 3, Tabla 4) deben remitirse al hospital para ser ingresados y darles un seguimiento más estrecho^15^, ^16^, ^17^, ^18^.

*Pacientes con enfermedad grave:*

1. Adultos con enfermedad clínica grave (p. ej., puntuación NEWS2 ≥ 5)^18^, que puede incluir síntomas significativos de neumonía, confusión/encefalitis y otras complicaciones graves (p. ej. infecciones cutáneas bacterianas secundarias graves o de difícil control, sepsis, proctitis, encefalitis, miocarditis, infecciones oculares o periorbitarias) (Tabla 3, Tabla 4).
2. Lesiones muy diseminadas, numerosas (≥100 lesiones) o necróticas.
3. Sospecha de infección corneal.
4. Dolor intenso y refractario por lesiones cutáneas.
5. Lesiones en zonas anatómicas con especial riesgo de cicatrización, estenosis u obstrucción que pueden afectar la orofaringe (causan disfagia o incapacidad para tragar o respirar); prepucio del pene, vulva, vagina, uretra o zona anorrectal (potencial de provocar estreñimiento o retención urinaria).

*Personas infectadas que suponen un riesgo para los contactos:*

1. Sujetos que conviven en un hogar con personas de riesgo (inmunocomprometidas, niños y embarazadas) y que no pueden reubicarse mientras que quien que dio positivo en la prueba se aísla por sí mismo.
2. Aquellos que es muy poco probable que puedan aislarse por sí mismos.

## Tratamiento

La mayoría de los pacientes con infección por MPOX no requieren tratamiento o bien es suficiente una terapia de síntomas leves^16^, ^17^, ^18^.

### Tratamiento de paciente con afectación leve o moderada sin criterios de ingreso

El tratamiento en los pacientes con afectación moderada que no necesitan ingresarse debe dirigirse principalmente al control de los siguientes aspectos.

#### Dolor^14^, ^16^

Lesiones orales o faríngeas: las úlceras orales o peribucales, las adenopatías regionales cervicales pueden interferir de forma importante con la nutrición por lo que el tratamiento del dolor es de suma importancia. En el brote actual en España, más de un tercio de los participantes presentaron complicaciones que requirieron analgésicos^10^. Las complicaciones más frecuentes fueron proctitis, amigdalitis, parafimosis por edema peneano y abscesos bacterianos. Se recomienda analgesia (paracetamol o antiinflamatorios no esteroideos por vía oral) o el uso de colutorio con lidocaína viscosa al 2% o gel al 1%^20^.

Lesiones cutáneas no sobreinfectadas: baños de asiento con suero/agua salina templada en el caso de heridas perineales o genitales.

#### Cuidado de la piel

Se debe evitar la infección de las lesiones con higiene adecuada o antisépticos tópicos, como la povidona yodada diluida.

El control de prurito a fin de evitar el rascado y el riesgo de sobreinfección se realiza preferentemente con antihistamínicos vía oral como loratadina 10 mg/24 h (máx. 40 mg/d) o hidroxicina 25 mg/24 h (máx. 50 mg/d).

Ante un caso de clara sobreinfección, se trata inicialmente con un antibiótico tópico (ácido fusídico o mupirocina). Si existe una infección más profunda (celutitis, erisipela) o superficial extensa se recurre a la antibioterapia por vía sistémica (tabla 5).

**Tabla 5:** Tratamiento antibiótico sistémico de lesiones cutáneas de la viruela del mono

Tratamiento elección	• Cefadroxilo oral, 1 g/12-24 horas durante 5 días • Cefalexina oral, 500 mg/8-12 horas, 5 días • Cloxacilina oral, 500 mg/6 horas, 5 días • Prolongar el tratamiento 10 días, si la evolución al tercer día no es adecuada
Tratamiento si hay alergia a penicilinas o sospecha de MARSA	• Clindamicina oral, 300-600 mg/8 horas durante 7 días • Trimetoprim/sulfametoxazol oral,160-800/12 horas, 5-10 días

Fuente: Heymann^17^.

MARSA: *Staphylococcus aureus* resistente a meticilina.

#### Afectación ocular^21^

Aunque esta es infrecuente, se debe valorar siempre porque la afectación corneal cursa con elevado riesgo de ceguera. Hay que instruir al paciente en la higiene de manos y evitar el contacto inadvertido con los ojos. Es conveniente asegurar que la afectación ocular es solo conjuntival antes de emplear antibióticos o antivirales tópicos. Los antisépticos tópicos (povidona iodada 0,6%), aún sin experiencia de uso, podrían ser de utilidad.

#### Nutrición^21^

En los pacientes debe asegurarse una correcta ingesta e hidratación, incluso con sales de rehidratación oral si fuese necesario. El uso de gel de lidocaína antes de las comidas puede ser de utilidad para facilitar la ingesta^14^.

#### Proctitis^21^

Para tratar las lesiones rectales o perianales, junto con el tratamiento analgésico habitual, puede utilizarse compuestos con sulfato de zinc o cobre (Pasta Lassar). La analgesia, generalmente del segundo escalón (opioides), deberá complementarse con laxantes a fin de evitar en lo posible el estreñimiento que puede exacerbar la clínica rectal. Debe valorarse el ingreso (ver apartado Criterios de derivación a atención hospitalaria) de los pacientes con clínica de proctitis que no responden al tratamiento de soporte.

#### Sobreinfección de lesiones cutáneas

Suponen el principal motivo de ingreso, especialmente por el riesgo de sepsis.

Si se sospecha una sobreinfección, se iniciará lo antes posible el tratamiento antibiótico sistémico (tabla 5)^22^.

### Tratamiento de los pacientes que requieren ingreso hospitalario^21^

Existen escasas opciones de tratamiento antiviral específico para la infección por MPOX.

• **Tecovirimat**, único antivírico aprobado como uso compasivo en humanos. Su empleo es muy limitado debido a existencias escasas y poco uso en humanos^16^, ^17^, ^23^, ^24^. El tratamiento es vía oral durante dos semanas.
  Es inductor del citocromo P450 (CYP)3A y CYP2B6, por lo que deberán valorarse potenciales interacciones con metadona, maraviroc, rilpivirina, darunavir o inhibidores de la fosfodiesterasa tipo 5 (PDE-5).
• **Brincidofovir**, no disponible por el momento.
• **Cidofovir**, ha demostrado actividad *in vitro* especialmente en el periodo presintomático, aunque debido a su escasa experiencia en humanos^25^, su uso no está aprobado en el momento actual.

El Ministerio de Sanidad, a través de la Federación de Asociaciones Científico Médicas (FACME) ha establecido las siguientes prioridades de tratamiento^21^:

• Prioridad 1: neumonía; encefalitis o meningoencefalitis; úlceras corneales u otras lesiones oculares con riesgo de secuelas permanentes que afecten a la visión y lesiones faríngeas que impidan la deglución de líquidos y/o presenten compromiso total o parcial de la vía aérea.
• Prioridad 2: proctitis graves; celulitis graves o con riesgo de secuelas permanentes; pacientes inmunodeprimidos con fiebre persistente o enfermedad diseminada con afectación generalizada.
• Prioridad 3: todos los demás pacientes infectados con MPOX que no tengan contraindicación.

## Medidas de prevención

### Medidas generales

La mayoría de los pacientes van a mantenerse durante la evolución de la enfermedad a nivel comunitario. Es importante cumplir con unas medidas generales y de prevención en el entorno domiciliario y sanitario (tabla 6). Los sujetos deben permanecer en aislamiento en su domicilio hasta que se haya resuelto toda la sintomatología, las lesiones cutáneas hayan desaparecido y las costras se hayan caído por completo. Además, debido al mayor riesgo de transmisión del brote actual relacionado con prácticas sexuales, se debe incidir en la necesidad de prácticas sexuales seguras (tabla 6).

**Tabla 6:** Medidas generales de prevención de la viruela del mono

**Medidas generales**	• Evitar el contacto cercano (piel con piel, incluido contacto íntimo o sexual) con cualquier persona que tenga una erupción similar a la viruela del mono • Evitar el contacto con animales o sus productos potencialmente infectados • Evitar el contacto con materiales que hayan estado en contacto con animales o personas enfermas • Aislar a los pacientes infectados • Higiene adecuada de las manos • Utilizar EPI adecuado en la atención a pacientes o en la manipulación de muestras para diagnóstico
**Control de la infección en ambientes domésticos**	• Aislamiento domiciliario del paciente (si no requiere ingreso hospitalario) evitando el contacto con otros miembros de la familia o mascotas, con baño separado (si es posible), autocuidado de sus lesiones y evitar la propagación de las mismas • Limitar el contacto cercano con otros miembros del hogar (mantener al menos un metro de distancia si el contacto es necesario) • Evitar especialmente el contacto con niños, embarazadas y personas inmunodeprimidas • Practicar por parte del enfermo la higiene de manos (los desinfectantes para manos a base de alcohol inactivan de manera eficiente el virus de la viruela del mono) y respiratoria (usar una máscara quirúrgica si hay síntomas respiratorios como dolor de garganta o tos en presencia de otras personas) • Cubrir las lesiones de la piel en la mayor medida posible (p. ej., mangas o pantalones largos, guantes) cuando esté cerca de otras personas o cuando se mueva fuera del área de aislamiento individual • Evitar el uso de lentes de contacto para prevenir una infección del ojo • Evitar afeitarse las áreas del cuerpo cubiertas por erupciones para impedir la propagación del virus • Limpieza y desinfección (p. ej. solución de hipoclorito de sodio al 0,1%), particularmente las superficies que se tocan con frecuencia, con productos de limpieza regulares/estándar • Lavar la ropa y la ropa de cama contaminadas con detergente regular a 60 °C • Usar una máscara quirúrgica por los convivientes cuando estén en contacto cercano con el paciente y guantes desechables cuando se toque directamente las lesiones • No compartir artículos domésticos ni artículos personales • Evitar cualquier actividad sexual durante el periodo de aislamiento. Tras la recuperación usar métodos de barrera (al menos 8-12 semanas después de que la erupción haya formado costras y estas se hayan caído)
**Control de la infección en el entorno sanitario**	• Habitación individual para paciente con sospecha o confirmación de viruela del mono • Limitar las visitas a lo estrictamente necesario para la atención y el bienestar del paciente • Los procedimientos que puedan provocar aerosoles (intubación, extubación, etc.), deben realizarse en salas con aislamiento y control del aire • Personal sanitario utilizar EPI (bata, guantes, protección ocular y mascarilla FFP2 o de nivel superior). • Manejo de ropa sucia según prácticas estándar recomendadas para el control de infecciones ambientales • La limpieza y desinfección deben realizarse con un desinfectante de uso hospitalario para patógeno viral emergente. El método de limpieza que se prefiere es el húmedo
**Prácticas sexuales seguras**	• Prácticas sexuales más seguras en HSH (principal modo de transmisión del brote actual) • Medidas: ∘ Descanso de comportamientos sexuales de riesgo hasta completar la vacunación contra la viruela del mono ∘ Limitar el número de parejas sexuales ∘ Evitar clubes y fiestas sexuales con contacto sexual anónimo con múltiples parejas ∘ Uso de métodos barrera (condones) ∘ Evitar besarse o intercambiar saliva ∘ Evitar el contacto piel con piel en actividad sexual ∘ Actividades sexuales sin contacto (virtuales) ∘ Lavar las manos, juguetes sexuales, ropa de cama, toallas, etc. después del sexo

Fuente: adaptada de Petersen et al.^9^

EPI: equipo de protección individual; HSH: hombres que tienen sexo con hombres.

### Profilaxis previa y posterior a la exposición mediante vacunación

La vacunación con la vacuna contra la viruela humana brinda protección contra la MPOX. Estas vacunas inducen una respuesta tanto humoral como celular que se dirige a una amplia gama de partículas virales y previene la replicación viral del virus de la MPOX. La infección o la inmunización con una vacuna otorga protección inmunológica cruzada contra otros virus del mismo género.

Durante el programa de erradicación de la OMS frente a la viruela se han desarrollado tres generaciones de vacunas. Inicialmente, se emplearon vacunas de primera generación basadas en diferentes cepas virales producidas en modelos animales con la presentación de reacciones adversas, desde erupción leve y fiebre, hasta cuadros más graves (*Vaccinia* progresiva y encefalitis posvacunal)^26^. Las de segunda generación se produjeron en cultivos celulares y provocaron niveles similares de respuesta inmunitaria, pudiendo ocasionar reacciones adversas graves (miocarditis, especialmente en inmunocomprometidos). Por último, se desarrollaron las de tercera generación basadas en cepas atenuadas con alteración en la replicación del virus *Vaccinia*, más seguras en población vulnerable^26^.

En la actualidad, la vacuna de tercera generación disponible es de virus vivo atenuado (deficiente en la replicación) de *Vaccinia* (MVA-BN). La vacuna fue aprobada inicialmente por la Administración de Alimentos y Medicamentos de los EE. UU. (FDA) en 2019 (bajo diferentes marcas [Jynneos®]) y posteriormente en Europa (Imvanex®) y Canadá (Imvamune®) para la inmunización activa contra la MPOX, la viruela y la enfermedad causada por el virus *vaccinia*^27^. Está indicada en la prevención de la MPOX en personas a partir de los 18 años con alto riesgo de infección y está contraindicada en alérgicas a las vacunas o sus componentes, aunque en una situación de exposición o brote, el riesgo de MPOX grave probablemente supere al de una reacción alérgica grave a la vacuna^28^. Se administran dos dosis (0,5 mL) separadas por cuatro semanas. La inyección es subcutánea y no requiere de aguja bifurcada especial (usada en las vacunas tradicionales)^25^, ^27^. No se ha informado de toxicidad cardiaca (miocarditis o pericarditis) con esta vacuna y, otros efectos adversos observados por la replicación de vacunas basadas en *vaccinia* (eczema *vaccinatum*, encefalitis posvacunal o *vaccinia necrosum*). La protección de la vacuna no se confiere hasta dos semanas después de recibir la segunda dosis. No se recomienda la administración a la población general. Se utiliza antes de la exposición «profilaxis preexposición» (PrEP) y después de la exposición «profilaxis posexposición» (tabla 7)^28^.

**Tabla 7:** Recomendaciones de vacunación contra la viruela del mono

**Profilaxis preexposición**	• Prácticas sexuales de alto riesgo, fundamentalmente, pero no exclusivamente GBHSH, incluidas dentro de las indicaciones de la profilaxis preexposición al VIH (PrEP) o con infección por el VIH en seguimiento y que no hayan pasado la enfermedad • Personas con riesgo ocupacional: ∘ Personal sanitario en atención de ITS/VIH que atienden a personas con prácticas de alto riesgo ∘ Personal de laboratorio que manejan muestras potencialmente contaminadas con MPOX ∘ Personal que se encarga de la desinfección de superficies en locales específicos donde se mantienen relaciones sexuales de riesgo, siempre que no se pueda garantizar otros medios de protección (EPI)
**Profilaxis posexposición**	• Contactos estrechos de casos confirmados con alto riesgo de enfermedad grave: ∘ Personas con inmunodepresión, incluyendo infección con VIH con ˂ 200 células/mL ∘ Mujeres embarazadas en cualquier trimestre de gestación ∘ Población infantil de cualquier edad • Personal sanitario que haya tenido un contacto cercano (inferior a 1 m en la misma habitación) sin EPI o que haya presentado alguna incidencia en el uso del EPI • Personal de laboratorio que maneje muestras de pacientes sospechosos o confirmados de MPOX que ha presentado alguna incidencia en el uso del EPI • Contactos estrechos (independientemente de su vulnerabilidad) de casos confirmados, en caso de mayor disponibilidad de dosis

Fuente: adaptada de SEFH^20^

EPI: equipo de protección individual; GBHSH: gays, bisexuales y hombres que tienen sexo con hombres. ITS: infecciones de transmisión sexual; MPOX: viruela del mono; PrEP: profilaxis preexposición; VIH: virus de inmunodeficiencia humana.

Si existe riesgo continuo por exposición a *Orthopoxvirus* más virulentos (virus *Variola* y virus de la MPOX), se recomienda una dosis de refuerzo cada dos años; si la exposición es a *Orthopoxvirus* menos virulentos (virus *Vaccinia* o de la viruela vacunal), una dosis de refuerzo al menos cada 10 años^28^.

## Financiación

Los autores de este artículo no han recibido recibido ningún tipo de apoyo financiero.

## Conflicto de intereses

Los autores no refieren ningún conflicto de interés.
